# INTERGENERATIONAL TRANSFERS AND HEALTH IN CHINESE OLDER ADULTS: DO RESILIENCE AND GENDER MATTER?

**DOI:** 10.1093/geroni/igac059.042

**Published:** 2022-12-20

**Authors:** Sizhe Liu, Wei Zhang, Keqing Zhang, Bei Wu

**Affiliations:** Shanghai University of Finance and Economics, Shanghai, Shanghai, China (People's Republic); University of Hawaii at Manoa, Honolulu, Hawaii, United States; University of Hawaii at Manoa, Honolulu, Hawaii, United States; New York University, New York, New York, United States

## Abstract

Emotional and instrumental supports from adult children have been shown to increase positive outcomes among older adults. In this study, we examined the association between intergenerational transfer and self-rated health as well as the mediating and moderating roles of resilience and gender, respectively, using data from Chinese older adults in Honolulu (N=400). We found that the impact of emotional and instrumental support varied by gender. Further, we found that resilience significantly mediated the positive effect of provided economic support on health of older men (resilience mediated 25.1% of the total effect) as well as the positive effect of received emotional support on health of older women (resilience mediated 35.3% of the total effect). Our study highlights the importance of considering resilience and gender differences when examining intergenerational transfers and health among Chinese older adults. The design of a culturally tailored policy would help promote the health of Chinese older adults.

